# Emergency One‐Stage Robotic Surgery for Congenital Biliary Dilatation With Bile Duct Perforation in a Pediatric Patient: A Case Report

**DOI:** 10.1111/ases.70122

**Published:** 2025-07-22

**Authors:** Hajime Asai, Chiyoe Shirota, Takahisa Tainaka, Satoshi Makita, Katsuhiro Ogawa, Masamune Okamoto, Akihiro Yasui, Shunya Takada, Kaito Hayashi, Yoichi Nakagawa, Daiki Katou, Hiroki Ishii, Ami Utsunomiya, Hiroo Uchida

**Affiliations:** ^1^ Department of Pediatric Surgery Nagoya University Graduate School of Medicine Nagoya Japan

**Keywords:** congenital biliary dilatation, perforation, robot‐assisted surgery

## Abstract

Bile duct perforation is a rare, serious complication of congenital biliary dilatation (CBD). While traditionally managed with a two‐stage surgical approach, recent reports suggest that one‐stage, minimally invasive approaches may be feasible. We present the case of a 13‐month‐old female who developed bile duct perforation associated with a protein plug in the common channel. Following the correction of coagulopathy, emergency robotic surgery was performed, including bile duct excision and Roux‐en‐Y hepaticojejunostomy. Despite significant adhesions and an anatomical anomaly in which the right hepatic artery coursed anterior to the common hepatic duct, robotic dissection and reconstruction were safely completed. The postoperative course was uneventful, and the patient was discharged. This report highlights the feasibility and potential advantages of one‐stage robotic surgery for CBD with perforation, even in the presence of active inflammation and vascular anomalies. Similar cases must be reported and evaluated to understand the indications and limitations of this approach.

## 1 Introduction

Bile duct perforation is a serious complication of congenital biliary dilatation (CBD) that necessitates prompt surgical intervention. Traditionally, the standard treatment approach involves a two‐stage surgical procedure: initial drainage to manage bile peritonitis, followed by definitive surgery once the acute inflammation subsides. However, this approach is associated with risks such as drain‐related complications, infection, and technical difficulties during the second operation due to adhesions and fibrosis [1]. Recently, one‐stage surgery has emerged as a feasible alternative, even in the presence of bile peritonitis [2]. Although early reports primarily described open surgical techniques [3], advances in minimally invasive techniques such as laparoscopic and robotic surgery have expanded the treatment options for CBD with bile duct perforation. These techniques offer potential benefits such as reduced invasiveness, improved visualization in inflamed or anatomically complex fields, and shorter recovery periods. Herein, we report the case of a pediatric patient with CBD and bile duct perforation that was successfully managed using emergency one‐stage robotic surgery.

## 2 Case Presentation

**FIGURE 1:**
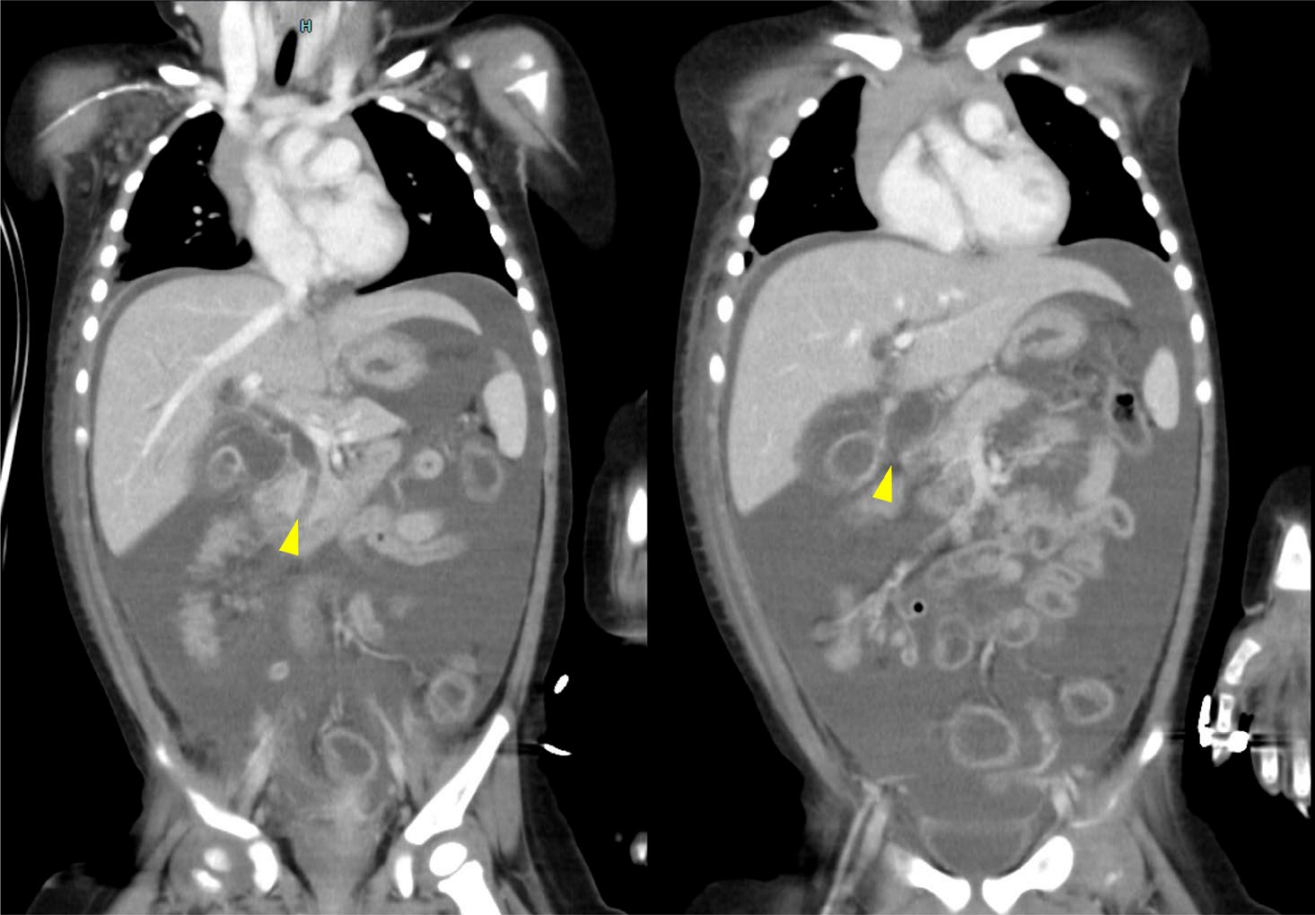
Preoperative contrast‐enhanced computed tomography. Contrast‐enhanced computed tomography (CT) revealed massive ascites, marked dilation of both intrahepatic and extrahepatic bile ducts, and a 19 mm‐long common channel containing a hyperdense structure suggestive of a protein plug (the left arrowhead). A discontinuity in the anterior wall of the common bile duct is indicative of perforation (the right arrowhead).

A 13‐month‐old female, weighing 11 kg, presented with abdominal pain and vomiting. The medical history of the patient was unremarkable. She was initially admitted to a local hospital with suspected acetonemic vomiting. Her symptoms worsened over 3 days, leading to abdominal guarding. Contrast‐enhanced computed tomography (CT) revealed massive ascites, dilation of the intrahepatic and extrahepatic bile ducts, and a 19 mm‐long common channel containing a hyperdense structure suggestive of a protein plug (Figure 1). A diagnosis of CBD (Todani classification type IV‐A) with bile duct perforation was made, and the patient was referred to our hospital for surgical intervention.

Upon admission, the patient was hemodynamically stable, except for tachycardia. Physical examination revealed abdominal distension and muscle guarding. Laboratory tests revealed mild systemic inflammation, elevated hepatobiliary enzyme levels, and coagulopathy (Table 1).

**TABLE 1:** The patient's vital signs and laboratory data on admission.

Vital signs		Complete blood count		Biochemical studies	
Body temperature	38.5°C	WBC	8200/μL	TP	5.3 g/dL
Heart rate	144/min	Neut	49%	Alb	3.3 g/dL
Blood pressure	120/71 mmHg	Lymp	45%	BUN	2.4 mg/dL
Respiratory rate	32/min	RBC	4 520 000/μL	Cre	0.15 mg/dL
SpO2	100%	Hgb	11.3 g/dL	AST	28 U/L
		Ht	34.5%	ALT	10 U/L
		Plt	299 000/μL	LDH	275 U/L
		Coagulation studies		ALP	395 U/L
		PT‐INR	1.49	γGTP	88 U/L
		APTT	93.5%	T‐Bil	1.8 mg/dL
		Fib	75 mg/dL	D‐Bil	0.8 mg/dL
		D‐dimer	39.8 μg/mL	Amy	508 U/L
		FDP	76.3 μg/mL	Lip	775 U/L
				CRP	1.57 mg/dL

**FIGURE 2:**
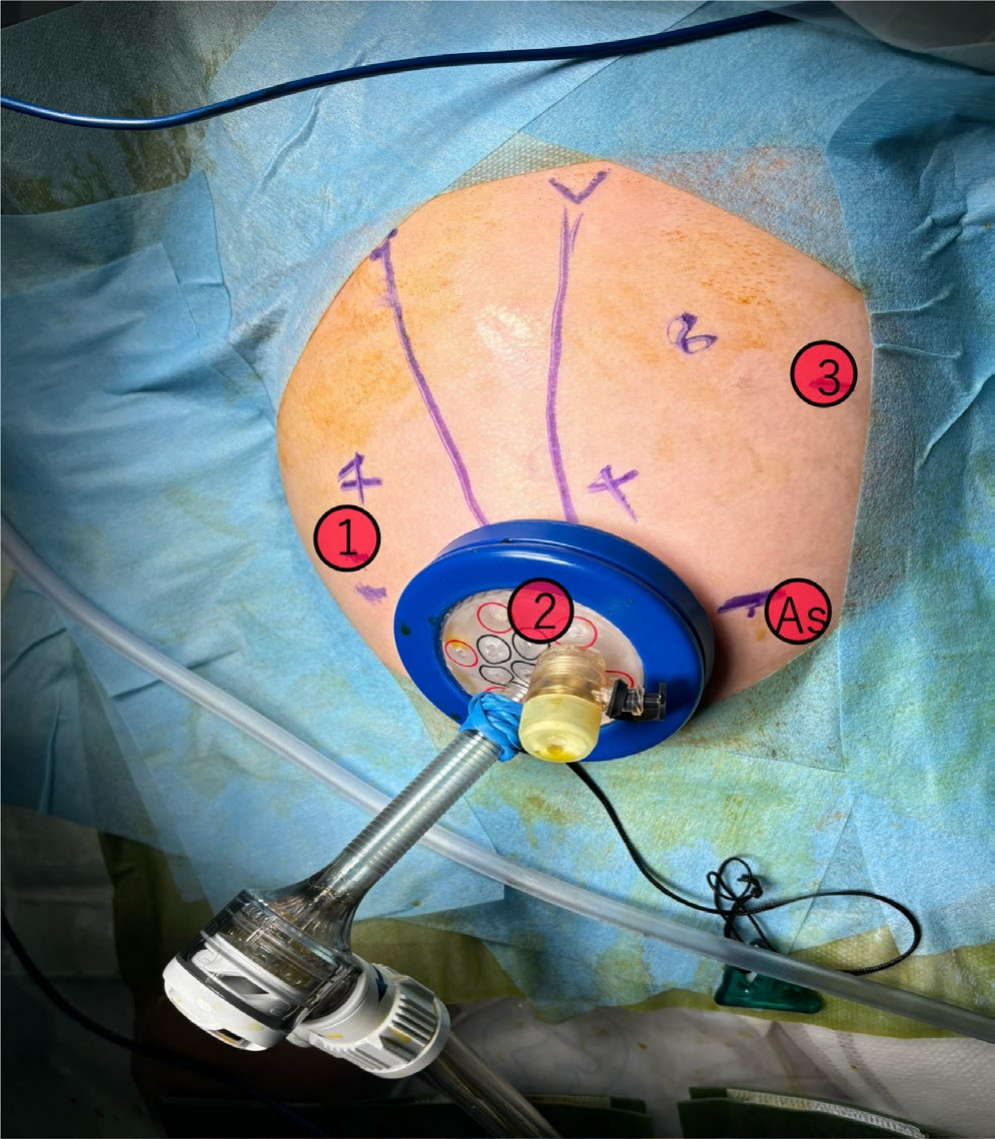
Port placement for robotic‐assisted surgery. Three robotic ports and one assistant port were strategically positioned as illustrated, following our standard protocol for robotic biliary surgery.

**FIGURE 3:**
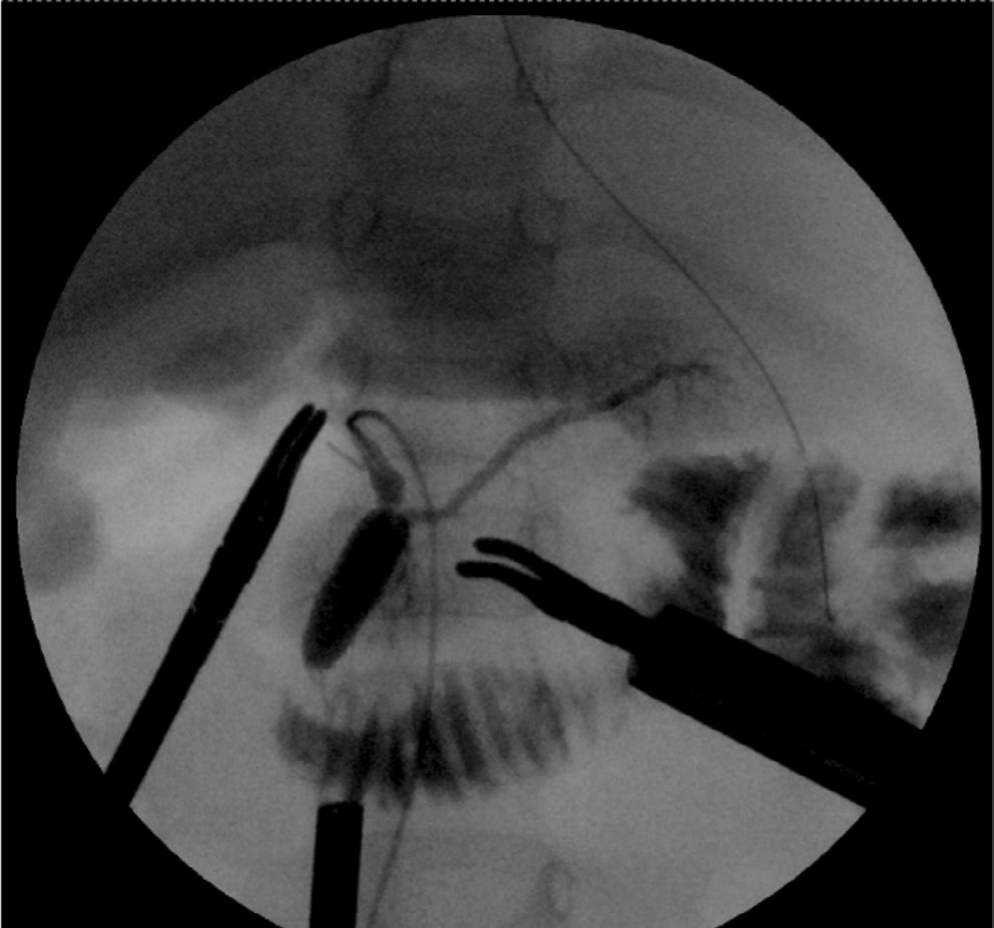
Intraoperative cholangiography. Intraoperative cholangiography revealed a protein plug within the common channel, resulting in poor contrast passage into the duodenum. Repeated irrigation improved contrast flow, although the protein plug remained partially visible.

Fresh frozen plasma was administered to correct coagulopathy immediately after admission, and the patient underwent emergency robotic surgery using the da Vinci Xi surgical system 6 h later. A Benz incision was made at the umbilicus to insert a multichannel port with additional robotic ports placed, following our conventional approach [4] (Figure 2). Bile‐stained ascitic fluid was observed. The hepatoduodenal ligament was inflamed and adhered to adjacent structures, including the duodenum, colon, and greater omentum. Initial adhesiolysis was performed laparoscopically, followed by robotic docking (Supporting Information: Video). Dissection of the common bile duct revealed a perforation on its anterior wall. The distal bile duct was mobilized into the pancreas without difficulty. Intraoperative cholangiography revealed a protein plug in the common channel with poor contrast passage into the duodenum, which improved after repeated irrigation (Figure 3). The bile duct was transected approximately 5 mm proximal to the common channel junction. Proximal dissection up to the hepatic hilum revealed no strictures. The right hepatic artery runs anterior to the common hepatic duct; the duct was successfully mobilized anteriorly to this vessel. A Roux‐en‐Y jejunal limb was prepared via an umbilical incision, and hepaticojejunostomy was completed robotically using 5–0 absorbable monofilament sutures with single knots via retrocolic route. A drain was placed behind the anastomotic site.

The total operative time was 337 min, with 50 mL of blood loss, including ascitic fluid. The postoperative course was uneventful. Oral intake was resumed on postoperative day 2, the drain was removed on day 6, and the patient was discharged on day 8. Follow‐up magnetic resonance cholangiopancreatography revealed no intrahepatic bile duct dilatation, but small residual protein plugs without pancreatic duct abnormalities. As the patient remained asymptomatic, follow‐up observation continued, with endoscopic retrograde pancreatography planned if the plug persisted with growth.

## 3 Discussion

Bile duct perforation is a rare but life‐threatening complication of the CBD. Historically, a two‐stage approach, initial drainage followed by definitive surgery, has been the standard treatment. In contrast, recent studies have reported that one‐stage surgery, including the laparoscopic approach, can be performed safely, even in cases of bile peritonitis, provided that appropriate surgical judgment is exercised [2, 3, 5]. This is the first case at our institution in which one‐stage robotic surgery was performed for CBD with bile duct perforation. However, since initiating laparoscopic procedures for CBD in 2013, we have treated four similar cases using one‐stage laparoscopic surgery. All were performed safely without perioperative complications. Based on this experience, we considered that robotic surgery could be safely attempted under stable general conditions.

Robotic surgery offers a minimally invasive alternative with enhanced visualization, tremor reduction, and improved dexterity, which are particularly valuable advantages in inflamed or anatomically complex operative fields. A meta‐analysis by Zhang et al. demonstrated that robotic‐assisted hepaticojejunostomy offers shorter anastomotic times and fewer complications than laparoscopic procedures [6]. Although our patient weighed only 11 kg, robotic surgery was safely performed. We previously demonstrated the safety and feasibility of robotic CBD surgery in patients weighing less than 10 kg [7].

Xie et al. recently conducted a retrospective study involving 64 pediatric patients with perforated choledochal cysts and showed robotic‐assisted surgery had outcomes comparable to open surgery and significantly fewer biliary complications than laparoscopic procedures [8]. However, in their cohort, one‐stage surgery was limited to patients with pseudocyst formation and no active bile peritonitis. In contrast, patients with complete perforation, as in our case, were treated using the conventional two‐stage procedure.

Despite marked inflammation and adhesions around the hepatoduodenal ligament, robotic‐assisted dissection and hepaticojejunostomy were safely completed. The right hepatic artery, which runs anterior to the common hepatic duct, was preserved without difficulty. These outcomes underscore the technical advantages of robotic assistance in the management of complex inflammatory conditions.

Although peritonitis is often considered a contraindication to primary anastomosis [9], our patient exhibited mild systemic inflammation and correctable coagulopathy. After stabilization with fresh frozen plasma, we concluded that one‐stage definitive surgery could be safely performed. The uneventful postoperative course supports previous reports suggesting that one‐stage procedures are feasible under selected conditions [2, 3].

To our knowledge, one‐stage CBD reconstruction in patients with systemic coagulation abnormalities has not been reported. In this case, despite the presence of coagulopathy, the patient's mild inflammatory response and stable condition allowed for safe radical intervention. Nonetheless, surgical indications must be carefully evaluated on a case‐by‐case basis. Based on our experience and current literature, one‐stage robotic surgery can be considered when the patient is hemodynamically stable, without severe infection or organ failure, and coagulopathy is correctable. Conversely, severe peritonitis, sepsis, or unstable general condition should be considered contraindications. Further accumulation of similar cases is warranted to clarify the indications and limitations of one‐stage robotic surgery under critical conditions.

## 4 Conclusion

This case demonstrates that one‐stage robotic surgery is a feasible and effective treatment option for CBD complicated by bile duct perforation, even in the presence of systemic inflammatory responses. Although careful case selection remains crucial, robotic surgery offers distinct advantages in the management of inflamed and anatomically challenging fields, enabling precise dissection and reconstruction with favorable outcomes.

## Author Contributions

Hajime Asai and Hiroo Uchida performed the surgery and prepared the manuscript. The remaining authors contributed to the clinical management and manuscript revision. All authors read and approved the final manuscript.

## Disclosure

Dr. Hiroo Uchida is an Editorial Board member of ASES Journal and a co‐author of this article. To minimize bias, they were excluded from all editorial decision‐making related to the acceptance of this article for publication.

## Ethics Statement

This study was conducted in accordance with the ethical standards of the Institutional and National Research Committee and the 1964 Declaration of Helsinki and its later amendments.

## Consent

Written informed consent was obtained from the patient's legal guardian for the publication of this case report and accompanying images.

## Conflicts of Interest

The authors declare no conflicts of interest.

## Supporting information


**Data S1.** Video. Summary of operative procedures.

## Data Availability

The data that support the findings of this study are available on request from the corresponding author. The data are not publicly available due to privacy or ethical restrictions.

## References

[1] S. Nennstiel , A. Weber , G. Frick , et al., “Drainage‐Related Complications in Percutaneous Transhepatic Biliary Drainage: An Analysis Over 10 Years,” Journal of Clinical Gastroenterology 49 (2015): 764–770. 25518004 10.1097/MCG.0000000000000275

[2] T. Ngoc Son , N. Thanh Liem , and V. Manh Hoan , “One‐Staged or Two‐Staged Surgery for Perforated Choledochal Cyst With Bile Peritonitis in Children? A Single Center Experience With 27 Cases,” Pediatric Surgery International 30 (2014): 287–290. 24463980 10.1007/s00383-014-3461-6

[3] G. Ohba , H. Yamamoto , M. Nakayama , S. Honda , and A. Taketomi , “Single‐Stage Operation for Perforated Choledochal Cyst,” Journal of Pediatric Surgery 53 (2018): 653–655. 28774506 10.1016/j.jpedsurg.2017.07.014

[4] T. Maeda , J. Liu , H. Uchida , et al., “Robotic Versus Laparoscopic Radical Surgery for Pediatric Congenital Biliary Dilatation: A Comparison of Surgical Outcomes of a Single Surgeon's Initial Experience,” Pediatric Surgery International 39 (2023): 261. 37660350 10.1007/s00383-023-05548-1

[5] T. Yin , S. Chen , L. Li , et al., “One‐ Versus Two‐Stage Single‐Incision Laparoscopic Cyst Excision and Hepaticojejunostomy in Patients With Completely Perforated Choledochal Cysts and Good Medical Conditions,” Pediatric Surgery International 38 (2022): 541–545. 35157126 10.1007/s00383-022-05073-7

[6] R. Zhang , S. Liu , T. Li , and J. Zhan , “Efficacy of Robot‐Assisted Hepaticojejunostomy and Laparoscopic‐Assisted Hepaticojejunostomy in Pediatric Congenital Choledochal Dilatation: A Systematic Review and Meta‐Analysis,” Pediatric Surgery International 39 (2022): 46. 36502451 10.1007/s00383-022-05286-w

[7] H. Ishii , C. Shirota , T. Tainaka , et al., “Safety and Feasibility of Robot‐Assisted Surgery for Pediatric Patients Weighing ≦ 10kg With Congenital Biliary Dilatation,” Journal of Robotic Surgery 19 (2025): 34. 10.1007/s11701-024-02181-5 39729146

[8] X. Xie , K. Li , and B. Xiang , “Surgical Outcomes of Robotic‐Assisted Cyst Excisions and Hepaticojejunostomies in Patients With Perforated Choledochal Cysts: A Single‐Center Retrospective Study,” Updates in Surgery 75 (2023): 571–580. 36441481 10.1007/s13304-022-01435-x

[9] S. Ojha , P. Agarwala , R. Sharma , and L. Bharadia , “Ruptured Choledochal Cyst: One‐Stage or Two‐Stage, Open or Laparoscopic Surgery?,” Journal of Minimal Access Surgery 19 (2023): 138–140. 36722538 10.4103/jmas.jmas_206_21 PMC10034790

